# Mechanisms of Skeletal Muscle Dysfunction in Cardiometabolic HFpEF and Its Reversal With Exercise Training

**DOI:** 10.1016/j.jacbts.2024.10.009

**Published:** 2024-12-23

**Authors:** Bharathi Upadhya, Dalane W. Kitzman

**Affiliations:** a Cardiovascular Medicine Section, Duke University School of Medicine, Durham, North Carolina, USA; b Department of Cardiovascular Medicine, Wake Forest University School of Medicine, Winston-Salem, North Carolina, USA

**Keywords:** branched-chained amino acids, exercise, heart failure with preserved ejection fraction, metabolism, mitochondria

**Figure:**
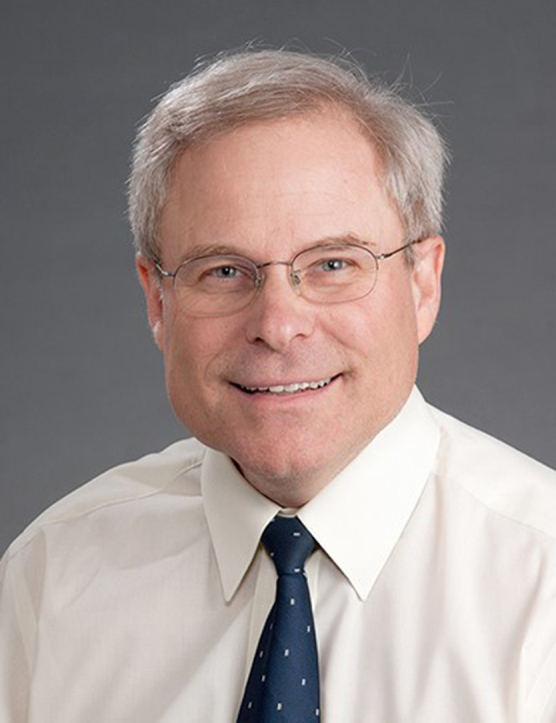

**Figure:**
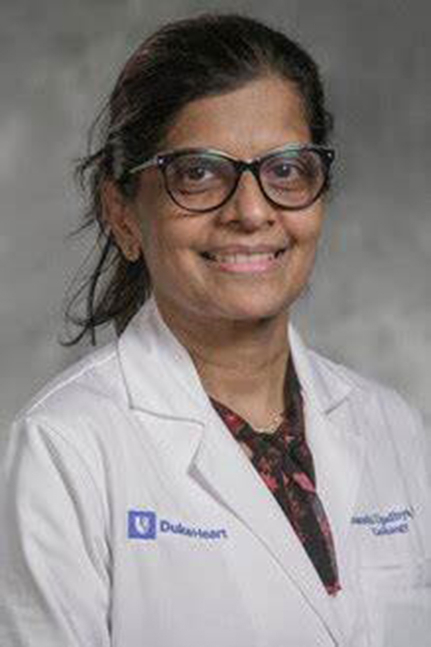

Severe exercise intolerance (EI) is the primary symptom in patients with heart failure with preserved ejection fraction (HFpEF), even when well compensated. EI is objectively measured as reduced peak oxygen consumption (VO_2peak_) and is a significant predictor of morbidity, mortality, and poor quality of life among patients with HFpEF.^1^ The mechanisms underlying the reduced VO_2peak_ in HFpEF are heterogeneous; the pathophysiology of EI is incompletely understood, and few effective therapies exist. Prior studies have identified both “central” impairment in cardiac output (CO) and “peripheral” impairment in skeletal muscle (SM) O_2_ conductance and utilization as critical drivers of reduced VO_2peak_ in HFpEF.^1^ Figure 1 summarizes the multiple abnormalities in SM structure and function that have been reported in patients with HFpEF and that likely contribute to their decreased EI.^1^, ^2^, ^3^, ^4^, ^5^, ^6^, ^7^ These abnormalities are likely caused by chronic inflammation, neurohormonal activation, and SM hypoperfusion and are intrinsic to the HFpEF syndrome rather than being a secondary consequence or an epiphenomenon.

**Figure 1:**
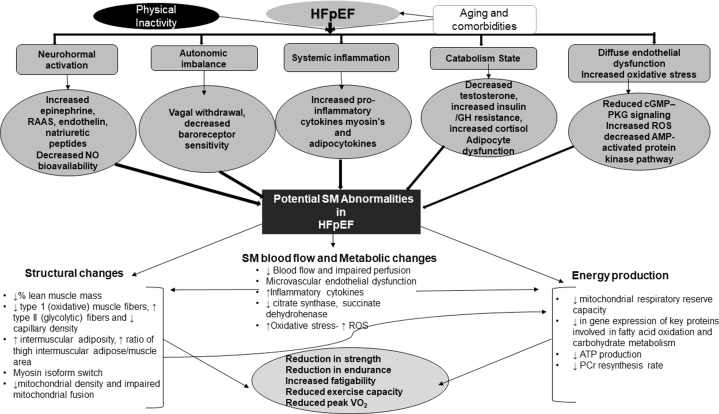
Potential Causes for the Skeletal Muscle Abnormalities in HFpEF AMP = adenosine monophosphate; ATP = adenosine triphosphate; cGMP = cyclic guanosine monophosphate; GH = growth hormone; HFpEF = heart failure with preserved ejection fraction; NO = nitric oxide; Pcr = phosphocreatine PKG = protein kinase; RAAS = renin angiotensin aldosterone system; ROS = reactive oxygen species; SM = skeletal muscle; VO_2_ = oxygen consumption.

Despite the importance of EI, few proven treatment options exist. Exercise training (ET) was the first strategy proven to improve EI,^1^ and multiple studies have confirmed that ET greatly improves EI and quality of life. Subsequently, weight loss via caloric restriction was shown to provide an additive benefit for ET in the large subgroup of patients with obesity-related/cardiometabolic HFpEF.^1^ However, despite its strong beneficial effects, the mechanisms of ET's benefit on EI are unknown.

Quiriarte et al^8^ conducted an elegant study to address this evidence gap in this issue of the *JACC: Basic to Translational Science*. They tested the hypothesis that ET may mitigate SM metabolic dysfunction in HFpEF. They created a model of cardiometabolic HFpEF using a 60% high-fat diet + N(ω)-nitro-L-arginine methyl ester in (0.5 g/L, pH to 7.4) supplemented drinking water to induce cardiometabolic HFpEF in 8-week-old male mice. Then, they randomly assigned the mice to a wheel running cage to engage in voluntary wheel running exercises or a standard cage with no running wheel for 5 weeks. Using a multi-omics approach, they demonstrated that this HFpEF model was associated with mitochondrial dysfunction and EI characterized by reduced branch chain amino acids catabolism (severe dysregulation substrate oxidation [leucine and palmitate]) and mishandling of fatty acid (incomplete fatty acid oxidation [FAO]). These metabolic defects and the associated EI were reversed mainly by ET. At the molecular level, the reversal of these effects appears to be predominantly related to branched-chain amino acids catabolic proteins. Thus, ET can rescue dysfunctional SM lipid and branched-chain amino acid oxidation and restore exercise capacity in mice with cardiometabolic HFpEF. The study supports that a further increase in gene expression and protein abundance appears to be involved in the improved metabolic capacity of SM to oxidize lipids.

The results of the present study further underscore the strong contribution of improvements in SM metabolism to the ET-related improvement in EI in patients with cardiometabolic HFpEF. Even though there was a dramatic improvement in exercise capacity with ET, there were no changes in cardiac hemodynamic function in response to ET, a phenomenon previously reported in patients with HFpEF, where >80% of the ET-related improvement was related to noncardiac factors.^9^ Further supporting the credibility of the strong contribution of SM dysfunction to EI and its improvement in HFpEF is that this pattern is also seen in HFrEF. Furthermore, among patients with mitochondrial myopathies, VO_2peak_ is decreased because of impaired oxidative metabolism in SM despite normal cardiac function. Indeed, studies show that SM abnormalities persist even when CO is relatively preserved and when CO is normalized with inotropes or cardiac transplantation.

These high-impact findings significantly extend and enhance our understanding of HFpEF and its central manifestation: chronic EI. Although the study has multiple layers of novel findings regarding cardiometabolic HFpEF, one of these, abnormal FAO as a critical underpinning of SM dysfunction in HFpEF, is particularly noteworthy given the dominance of the obesity-related phenotype of HFpEF. The authors’ results build upon a prior study, which showed that increased circulating metabolites reflecting impaired or dysregulated FAO were significantly elevated in HFpEF and heart failure with reduced ejection fraction (HFrEF) compared with no-heart failure control subjects. These metabolites were significantly higher in HFrEF than HFpEF, increasing linearly with declining LVEF.^5^ This highlights that dysregulated FAO might be a specific metabolic pathway in heart failure syndrome regardless of LVEF status.

When muscle repeatedly contracts for long periods, the ATP supply needs to be constantly replenished through mitochondrial oxidative phosphorylation. If the intricate metabolic pathways within the mitochondria become altered and less efficient, endurance within the SM decreases. The rate of breakdown and resynthesis of high-energy phosphates during and following exercise are fundamental determinants of whole-body VO_2_ during exercise and recovery. As the sole mechanism for utilizing oxygen and fuel substrate to produce energy, mitochondrial health is a critical determinant of VO_2peak_. Our group and others showed that patients with HFpEF have a decreased number of type I oxidative fibers; rapid depletion of high-energy phosphate early during exercise; smaller mitochondria; decreased SM mitochondrial content; reduced proteins and complexes that are involved in energy fuel metabolism; decreased oxidative capacity as reported by citrate synthase activity; the expression of mitochondrial structural proteins; and marked abnormalities in SM mitochondrial function that were significantly associated with their reduced exercise capacity, muscle fatigue, and muscle strength.^1^, ^2^, ^3^, ^4^, ^5^, ^6^, ^7^

As previously said, marked baseline SM dysfunction, which plays a significant role in EI and prominently contributes to improved exercise capacity following ET, is not new. It is also seen in classic HFrEF.^1^ Although the improvement in VO_2peak_ with ET is due in part to “noncardiac” peripheral adaptations (eg, improved SM) in both HFrEF and HFpEF, the relative contribution of peripheral adaptations to improvements in EI appears to be much more prominent in patients with HFpEF. Our group showed that 84% of the improvement in VO_2peak_ after 4 months of ET was attributed to increases in peak exercise a-vO_2diff_, with little effect on peak exercise CO.^9^ Similarly, Fu et al^10^ showed that 12 weeks of high-intensity interval training improvements in VO_2peak_ being exclusively driven by increased estimated peak exercise a-vO_2diff_ with no changes in estimated peak exercise stroke volume and cardiac index. In an animal model of HFpEF, Bowen et al^6^ found multiple SM abnormalities, including reduced in situ mitochondrial respiratory reserve capacity, a key measure of SM oxidative phosphorylation ameliorated with ET. Winzer et al^7^ showed in their substudy that SK mitochondrial enzyme activity (citrate synthase and mitochondrial complex-I) and expression (mitochondrial complex I, II, and IV) were increased significantly following 3 months of supervised ET and associated with improved EI. Taken together, SM oxidative capacity and efficiency can be improved by ET, which increases capillary and mitochondrial density, changes muscle fiber subtypes distribution, and increases red blood cell capillary transit time through the SM vasculature.^1^

In summary, these data from Quiriarte et al^8^ significantly extend our understanding of EI in cardiometabolic HFpEF and support the critical importance of abnormal metabolism and mitochondrial function. Mitochondrial abnormalities are linked to many common disease processes and are potentially modifiable by pharmacological and behavioral interventions, including diet and exercise, highlighting their potential as future specific therapeutic targets.

## Funding Support and Author Disclosures

Supported in part by the Kermit Glenn Phillips II Chair in Cardiovascular Medicine and National Institutes of Health grants: U01AG076928, R01AG078153, R01AG045551, R01AG18915, P30AG021332, U24AG059624, and U01HL160272. Dr Kitzman has been a consultant for AstraZeneca, Pfizer, Corvia Medical, Rivus, Boehringer Ingelheim, Novo Nordisk, Rivus, and St. Luke’s Medical Center; has received grant support from Novartis, AstraZeneca, Bayer, Pfizer, Novo Nordisk, Rivus, and St. Luke’s Medical Center; and owns stock in Gilead Sciences. Dr Upadhya has received support from Novartis and Corvia.
